# Theoretical Boundaries of Annual Flood Risk for Single-Family Homes Within the 100-Year Floodplain

**DOI:** 10.1007/s41742-024-00577-7

**Published:** 2024-03-15

**Authors:** Ayat Al Assi, Rubayet Bin Mostafiz, Carol J. Friedland, Robert V. Rohli

**Affiliations:** 1 Department of Biological and Agricultural Engineering, LaHouse Research and Education Center, Louisiana State University Agricultural Center, Baton Rouge, LA 70803 USA; 2 Bert S. Turner Department of Construction Management, Louisiana State University, Baton Rouge, LA 70803 USA; 3 Coastal Studies Institute, Louisiana State University, Baton Rouge, LA 70803 USA; 4 Department of Oceanography & Coastal Sciences, College of the Coast & Environment, Louisiana State University, Baton Rouge, LA 70803 USA

**Keywords:** Flood risk, 100-year floodplain, A Zone, Synthetic parameters, Average annual loss (AAL), Mitigation strategies

## Abstract

Special flood hazard areas (SFHAs), defined as having an annual probability of occurrence of 1 percent or above, are used by U.S. Federal Emergency Management Agency (FEMA) to demarcate areas within which flood insurance purchase is required to secure a mortgage. However, quantifying flood risk within SFHAs can be challenging due to the lack of modeled flood depth data for all return periods. To address this issue, this research quantifies flood risk indicated by average annual loss (AAL) within the A Zone—the subset of the SFHA where wave heights can potentially range from 0 to 3 feet. The methodology resolves the Gumbel quantile function for four distinct flooding cases (i.e., locations flooded at return periods exceeding 1.58-, 10-, 25-, and 50-year return period events) and generates synthetic flood hazard parameters for these cases within the 100-year floodplain, as well as with additional elevation above the base flood elevation (BFE), known as freeboard, for single-family homes with different attributes. The results indicate that for single-family homes in the A Zone, with the lowest floor elevated to the BFE, the AAL ranges from 0.3 to 1 percent of the building replacement cost value. Adding one foot of freeboard reduces flood risk by over 90% if the annual flood risk is between the minimum and 25th percentiles and the 100-year flood depth is less than two feet. The demonstrated approach helps enhance flood resilience in the A Zone, demonstrating the feasibility of proactive measures to protect communities.

**Supplementary Information:**

The online version contains supplementary material available at 10.1007/s41742-024-00577-7.

## Highlights

- Quantifying flood risk is challenging due to unavailability of flood depth data.
- The synthetic flood parameter approach generates a library of synthetic flood parameters that is used to assess flood risk.
- Elevating the lowest floor of a home leads to reducing flood risk substantially.
- Flood risk assessment contributes to enhancing flood resilience and guiding proactive measures to protect communities in flood-prone zones.

**Supplementary Information:**

The online version contains supplementary material available at 10.1007/s41742-024-00577-7.

## Introduction

Floods are among the costliest natural disasters worldwide and in the U.S.A., leading to significant loss of human life and property when effective risk assessment and mitigation strategies are lacking (Fan and Davlasheridze 2016; Mostafiz et al. 2022a; Pakhale et al. 2023; Petrović et al. 2021; Pricope et al. 2022). Between 1980 and 2023, the U.S.A. was affected by 44 catastrophic floods that caused a total of $196.6 billion (consumer price index adjusted) in direct losses (NOAA 2023). In upcoming years, flood-related property damage may increase due to climate change (Sastry 2022) and sea level rise (Mrozik 2022). Consequently, researchers across the globe have focused on flood risk and loss assessments.

In the United States, flood risk regions and base flood elevation (BFE), which is equivalent to the 100-year flood elevation, have been determined using floodplains derived from the Federal Emergency Management Agency (FEMA; Xian et al. 2015). The 100-year floodplain, also known as the special flood hazard area (SFHA), has been utilized as an indicator for identifying high-risk flood zones due to its likelihood of having an annual probability of occurrence of 1 percent or above, and being labeled on a Flood Insurance Rate Map (FIRM) with the letters “A” or “V.” Areas beginning with “A” (i.e., A, AO, AE, and A1–A30, A99) refer to the subset of the SFHA that is not considered part of the Coastal High Hazard Area, while those beginning with “V” (i.e., V, VE, V1–30) denote the Coastal High Hazard Area, which experiences high-velocity waves. Despite SFHA regulations (Hamstead et al. 2021), properties within the SFHA still face substantial flood risk. Surprisingly, recent assessments show a substantial difference between the estimated 41 million people residing in this high-risk zone and FEMA's identification of only 13 million within the 1% annual chance floodplain (Association of State Floodplain Managers 2020). This discrepancy continues with an average annual loss (AAL) of $13.2 billion in the U.S.A., with projected increases of 33.8% by 2050 (Wing et al. 2022).

In recent years, much attention has been devoted to studying flood hazards within the SFHA (Blessing et al. 2017; Gori et al. 2019; Habete & Ferreira 2017; Johnston and Moeltner 2019; Ludy and Kondolf 2012; Mobley et al. 2021; Posey & Rogers 2010; Prasad 2016; Rath et al. 2018; Shu et al. 2022; Smiley 2020). Despite these efforts, only limited consideration has been given to quantifying the flood risk to residential structures in the SFHA, highlighting the need for further research to understand residential flood risk to shape future policies. In the U.S.A., Pistrika and Jonkman (2010) examined the relationship between flood features and residential building risk in post-Hurricane-Katrina New Orleans. Their study utilized hydrodynamic flood simulations and a large data set of approximately 95,000 residential buildings to analyze the effects of various sources of flooding on buildings. An examination of inland and coastal flood risk for single-family residences in two Texas counties led Czajkowski et al. (2013) to conclude that wide variations in within-county flood risk exist and that substantial exposure to storm surge occurs beyond designated risk areas. In New Jersey, Armal et al. (2020) quantified flood risk within the SFHA for residential buildings using parcel-level flood risk data output by the First Street Foundation Flood Model. Al Assi et al. (2023a) evaluated neighborhood-scale flood risk in Metairie, Louisiana, utilizing a refined numerical integration method to quantify AAL for building, contents, and use, and for different owner/occupant types. Lüdtke et al. (2019) used a case study approach to probabilistic flood loss modeling for computing flood losses to residential buildings in Europe. In Dhaka, Bangladesh, Chen et al. (2016) evaluated direct risk to life and health using hydraulic modeling and object attributes. While these studies provide valuable insights into the effects of floods on residential buildings and can inform policy decisions aimed at mitigating flood damage, they are all location specific and dependent on flood and building attribute data availability.

Evaluating flood risk involves assessing flood occurrence probability and its consequences. Previous studies have used AAL as an expression of annual flood risk (Al Assi et al. 2023b; Bowers et al. 2022; Friedland et al. 2023; Gnan et al. 2022a, b; Hallegatte et al. 2013; Mostafiz et al. 2022b), which takes into account the costs associated with the building itself and its contents, as well as indirect costs such as use risk during renovation (Al Assi et al. 2023a). The Gumbel distribution is commonly used for flood peak prediction and flood frequency analysis (Mostafiz et al. 2022c; Parhi 2018; Patel 2020; Singh et al. 2018). In predicting annual flood risk, the Gumbel parameters play a crucial role as they describe the relationship between flood depth ($d$) and the double natural logarithm of the non-exceedance probability ($P$). However, the use of this algorithm requires flood depth data for at least two different return periods, making site-specific flood risk quantification challenging in 100-year floodplains where hydraulic analysis and flood depth data may be limited.

Flood mapping is typically carried out by FEMA, whose models are considered the gold standard for understanding flood hazards at a local scale. However, replicating these models at a continental scale requires significant resources and labor, and FEMA has only modeled one-third of U.S.A. rivers since the national flood mapping program began in 1967. Additionally, only one-fourth of these models have been updated in the past 5 years (Association of State Floodplain Managers 2020; Wing et al. 2022). This disparity and the aging quality of FEMA's flood models, especially their limited coverage of smaller catchments, echo similar challenges found in hazard maps used for broader risk assessments globally or continentally, thereby hindering precise risk calculations. Although FEMA’s Risk MAP program has attempted to offset these shortcomings by introducing non-regulatory tools like flood depth grids aimed at aiding communities in flood risk reduction since 2012, the inconsistent availability of these resources across different communities persists (U.S. Government Accountability Office 2021). Given the immense challenge of rapidly improving flood mapping data, a new approach that is independent of location and yields generalized annual flood risk is urgently needed.

Recognizing the limitations and flood modeling issues, recent studies worldwide, such as those by Carozza and Boudreault (2021), Dottori et al. (2022), Karamouz et al. (2008, 2010), Sarkar and Mondal (2019), and Zzaman et al. (2021), have made significant progress in flood modeling. Within the United States, Bates et al. (2021) have made efforts to improve the accuracy of broad-scale flood inundation models, offering a comprehensive national-scale analysis of flood hazards and insights into the potential impacts of changing flood conditions on land development and existing defense measures. Wing et al. (2021) adapted the Bates et al. (2021) model, providing validation of a continental-scale flood inundation model and introducing a framework for modeling historical flood events. Recently, Wing et al. (2022) utilized data published by Bates et al. (2021) to estimate national flood risk and its demographic spread, expanding the applicability of the models globally for enhanced flood risk management. However, identifying flood risk at specific locations and individual levels, crucial for raising awareness and implementing adaptive flood mitigation measures, remains a gap.

Despite the efforts in assessing flood hazards, conducting flood modeling, and evaluating residential flood risk within the SFHA, a critical challenge continues in identifying specific locations and individual-level flood risk. This poses a critical obstacle in enhancing building resilience and raising individual awareness. Emphasizing the value of mitigation strategies at the individual level becomes vital for enhancing awareness among homeowners of flood risk. Effectively addressing this challenge needs a new approach that is independent of location and provides generalized annual flood risk.

This paper aims to overcome these challenges in characterizing flood risk within the A Zone through a systematic approach. Specifically, this study sets out two main objectives: (1) provide a meaningful estimate of the range of expected annual flood risk in the A Zone, and (2) calculate the reduction in annual flood risk via elevation for homes in the A Zone. The lack and limited flood hazard data in the A Zone are addressed by developing a library of combinations of synthetic, regression-derived Gumbel parameters that meet the mathematical definition of the A Zone.

Therefore, this paper addresses the challenges in characterizing flood risk in the A Zone. The approach resolves the Gumbel quantile function for four distinct flooding cases (i.e., location flooded at return periods exceeding 1.58-, 10-, 25-, and 50-year return period events), generating a library of synthetic flood parameters that meet the flood conditions in the A Zone. The method is used to assess the flood risk for hypothetical single-family homes with various features (i.e., one vs. two-plus stories, with vs. without basement) located in the A Zone in the United States. This study also explores the relative reduction in flood risk achieved with each additional first-floor elevation (FFE) increment (i.e., one to four feet) above the BFE (i.e., freeboard). To validate and demonstrate the utility of the flood risk assessment generated here, real flood parameters are derived from available flood depth data at multiple return periods from various locations in California, Colorado, Michigan, New Hampshire, New Jersey, and Oregon. The results of the flood risk assessment using these real flood parameters are compared to those generated using the synthetic parameters.

The contribution of this research lies in its establishment of a unique, stable, and generalized approach to define flood hazards within the A Zone. These parameters are applied to assess single-family home flood risk in the A Zone, clarifying the quantifiable advantages of implementing freeboard strategies. The methodology and results benefit various stakeholders, including homeowners, developers, and other partners needing to understand flood risk and enhance flood resilience through risk-informed construction techniques. This will improve homeowners’ awareness, allowing them to have a clearer understanding of their property's flood risk, and empowering them to make informed decisions regarding protective elevation strategies for their homes. Developers will utilize this information to enhance building resilience, and insurance companies use it as input for adjusting policies, potentially leading to reduced risks and premiums.

## Methodology

The methodology to generate synthetic flood parameters consists of three steps. First, the Gumbel quantile function is resolved using the mathematical definition of A Zone flooding. Second, distinct flooding cases (i.e., location flooded at return periods exceeding 1.58, 10, 25, and 50 years) are defined to further delineate flood risk using potentially available flood data. Finally, a library of synthetic flood parameters is generated through the definition of the range of each flood parameter and by resolving the ratio of flood parameters for each flood case. Using the library of synthetic characteristics and a new computational framework (Al Assi et al. 2023a), AAL for each case and the reduction with additional elevation above BFE are computed for a hypothetical single-family home with one vs. two-plus stories, and with vs. without basement, located in the A Zone. The validity of the results is confirmed by comparing AAL generated from synthetic parameters with that generated from real data in various locations in the United States.

### Gumbel Distribution

The Gumbel distribution function, as described by Eqs. (1–3), provides the most suitable representation for the relationship between return periods and the corresponding flood depths above the ground ($d$) (Gnan et al. 2022c; Mostafiz 2022; Singh et al. 2018). Equation (1) demonstrates the cumulative distribution function (CDF) associated with the Gumbel distribution, which quantifies the annual probability of non-exceedance ($p$; Al Assi et al. 2023a, b; Gnan et al. 2022c). By solving Eq. (1), Eq. (2) establishes the relationship between $d$ and the double natural logarithm of $p$. The Gumbel distribution parameters (i.e., regression coefficients), $u$ and $a$, referred to as regression coefficients, determine the y-intercept and slope in this relationship. Finally, Eq. (3) expresses $p$ by flood return period ($T$).

1 $$F\left( d \right) = p\left( {X \le d} \right) = \exp \left[ { - {\text{exp}}\left( { - \left( {\frac{d - u}{a}} \right)} \right)} \right],$$

2 $$d = u - a\ln \left[ { - \ln \left( p \right)} \right],$$

3 $$p = 1 -{ \frac{1}{T}}.$$

In typical studies, the $u$ and $a$ flood parameters are determined using the Gumbel distribution at a specific location, requiring flood depths corresponding to at least two return periods. As this study seeks to overcome the location dependence challenge, synthetic flood parameters are generated based on the theoretical definition of flooding in the A Zone and further refined through flood cases.

To determine the relationship between flood depth within a building ($dh$) and the resulting percentage damaged, depth-damage functions (DDFs) are employed (Mostafiz et al. 2021). The $dh$ is determined by subtracting the FFE from $d$, as illustrated in Eq. (4) (Gnan et al. 2022c; Al Assi et al. 2023a). Here, $d$ is calculated by 0.5-foot $dh$ increment, using Eq. (4). The flood parameters and $d$ are then utilized to compute the associated exceedance probability ($P$) using Eq. (2) and Eq. (5). Consequently, the selected DDFs are transformed into a function of $P$ using relationships outlined in Eqs. (1–5):

4 $$dh = d - FFE,$$

5 $$P = 1 - p.$$

### Flood Case Definition

Because the A Zone is subject to an annual probability of occurrence of 0.01 or above, the flood depth in the A Zone exceeds zero when a flood having a 100-year return period occurs. As the boundary of the SFHA delineates the one percent annual chance flood event, locations within the SFHA may be prone to inundation when 10-, 25-, and 50-year return period floods occur. The process of generating synthetic $u$ and $a$ begins by substituting $p$ from Eq. (3) into Eq. (2) (represented as Eq. 6), for the 100 (i.e., *T*)-year return period, assuming that $d$ exceeds zero (Eq. 7):

6 $$d = u - a\ln \left[ { - \ln \left( {1 - {\frac{1}{100}}} \right)} \right],$$

7 $$0 < u - a\ln \left[ { - \ln \left( {1 - {\frac{1}{100}}} \right)} \right].$$

Given that flood depth grids are most often available for 10-, 25-, 50-, 100-, and 500-year return periods, multiple flooding cases exist for return periods less than 100 years within the A Zone. In this study, four cases are considered, assuming that the 100-year return period event floods all A Zone locations in all four cases. A second assumption differs based on the flooding case. Specifically, case 1, 2, 3, and 4 locations are assumed to flood at return periods exceeding 1.58 (i.e., the minimum allowable return period), 10, 25, and 50 years, respectively (Eq. 8).

8 $$0 \ge u - a {\text{ln}}\left[ { - \ln \left( {1 - {\frac{1}{T}}} \right)} \right]\left\{ {\begin{array}{*{20}c} { T = 1.58 \;\;{\text{for }}\;{\text{Case}} 1} \\ {T = 10 \;\;{\text{for }}\;{\text{Case }}2} \\ {T = 25\;\; {\text{for}} \;C{\text{ase}} 3} \\ {T = 50 \;\;{\text{for}} \;C{\text{ase}} 4} \\ \end{array} .} \right.$$

### Synthetic Flood Parameters

To generate the synthetic flood parameters, it is necessary to determine the bounds of the ratio between the flood parameters $u$ and $a$ in the A Zone to provide the range of this ratio at each flooding case. This facilitates the identification of all potential combinations that meet the criteria for each flooding case. By simplifying Eqs. (7, 8), the ratio between flood parameters $u$ and $a$ is obtained. Solving Eq. (7) equal to zero establishes the minimum limit for this ratio across all flooding cases (Eq. 9), while solving Eq. (8) provides the upper limits for the ratio specific to each flooding case (Eq. 10):

9 $${\frac{u}{a}} > - 4.600,$$

10 $${\frac{u}{a}} \le \left\{ {\begin{array}{*{20}c} { - \;0.002, \;T = 1.58\;\;{\text{ for}}\;{\text{ Case 1}}} \\ { - \;2.250, \;T = 10\;\; {\text{for }}\;{\text{Case}} 2} \\ { - \;3.200, \;T = 25 \;\;{\text{for}}\;{\text{ Case}} 3} \\ { - \;3.900, \;T = 50 \;\;{\text{for}}\;{\text{ Case}} 4} \\ \end{array} } \right..$$

Equation (11) shows the ratio of the flood parameters in locations flooded at return periods exceeding 1.58, 10, 25, and 50 years, respectively:

11 $$- \;4.600 < {\frac{u}{a}} \le \left\{ {\begin{array}{*{20}c} { - \;0.002, \;T = 1.58\;\;{\text{ for}}\;{\text{ Case}} 1} \\ { - \;2.250, \;T = 10 \;\;{\text{for}}\;{\text{ Case }}2} \\ { - \;3.200, \;T = 25 \;\;{\text{for }}\;{\text{Case }}3} \\ { - \;3.900, \;T = 50 \;\;{\text{for}}\;{\text{ Case}} 4} \\ \end{array} .} \right.$$

After defining the range of the $u$ to $a$ ratio for each flooding case, the bounds of each individual parameter must be determined. By definition, flood parameter $a$ must exceed zero because flood events of longer return periods always have greater flood depth than events at shorter return periods. Likewise, $u$ must be negative, as indicated by Eq. (11), which is consistent with the assumption made by Mostafiz et al. (2022c) that $u$ is positive for water bodies or coastal areas and negative for terrestrial locations such as residential areas. Al Assi et al. (2023c) determined the maximum value of $a$ to be 4.60, calculated by analyzing the flood depths of multiple return periods in 13 counties along the Atlantic and Gulf coasts, assuming that the upper limit of this parameter occurs in coastal areas. By utilizing the upper limit of $a$ (i.e., 4.60) and the ratio between $u$ and $a$ defined in Eq. (11), the upper limit of $u$ is determined for each flooding case.

Initially, all possible combinations of $u$ and $a$ within their acceptable ranges are considered, at increments of 0.1, with the goal of describing the relationship between $d$ and the double natural logarithm of $p$. However, any combination of $u$ and $a$ yielding a ratio falling beyond the acceptable range specified in Eq. (11) is eliminated. The remaining combinations of $u$ and $a$ are utilized to determine $d$ at 1.58-, 10-, 50-, 100-, and 500-year return periods, as defined in Eq. (2). A plot of $d$ vs. the double natural logarithm of $p$ is generated from these calculations. This plot confirms the assumption for each flooding case.

### Annual Flood Risk and Risk Reduction

Annual flood risk, represented as AAL, is calculated by integrating flood loss by the annual probability of exceedance over all possible probabilities, as defined in Eq. (12) (Al Assi et al. 2023a, c; Gnan et al. 2022c),

12 $${\text{AAL}} = \mathop \smallint \limits_{{P_{{{\text{min}}}} }}^{{P_{{{\text{max}}}} }} L\left( P \right){\text{d}}P,$$

where $P_{{{\text{min}}}}$ is the lowest annual exceedance probability and $P_{{{\text{max}}}}$ is the highest exceedance probability. $L\left( P \right)$ includes losses that are proportional to replacement cost value ($V_{{\text{R}}}$; Kodavatiganti et al. 2023) of the building and its contents ($L_{{\text{B}}}$ and $L_{{\text{C}}}$, respectively) as well as use loss ($L_{U}$) represented as the number of months that the structure is out of service. Therefore, AAL is computed for building (${\text{AAL}}_{{\text{B}}}$) and contents (${\text{AAL}}_{{\text{C}}}$) as a proportion of $V_{{\text{R}}}$, and for use (${\text{AAL}}_{{{\text{use}}}}$). The ${\text{AAL}}_{{\text{B}}}$ and ${\text{AAL}}_{{\text{C}}}$ are then converted to dollar figures by multiplying them by $V_{{\text{R}}}$ as shown in Eqs. (13–15). The ${\text{AAL}}_{{{\text{use}}}}$ for the homeowner is multiplied by the monthly rent borne by homeowner ($R_{{\text{l}}}$), assuming that rent for one year is one-seventh of $V_{{\text{R}}}$ (Amoroso et al. 2008; Eqs. (16, 17)).

13 $$V_{{\text{R}}} = C_{{\text{R}}} \times A,$$

14 $${\text{AAL}}_{{\text{B}}\$} = {\text{ AAL}}_{{\text{B}}} {{ \times }}V_{{\text{R}}} ,$$

15 $${\text{AAL}}_{{\text{C}}\$} = {\text{ AAL}}_{{\text{C}}} \times V_{{\text{R}}} ,$$

16 $${\text{AAL}}_{{\text{U}}\$} = {\text{ AAL}}_{{{\text{use}}}} \times R_{{\text{l}}} {,}$$

17 $$R_{{\text{l}}} = {\frac{{V_{{\text{R}}} }}{{84 \;{\rm {months}}}}}.$$

The total AAL (${\text{AAL}}_{{\text{T}}\$}$) which is the sum of ${\text{AAL}}_{{\text{B}}\$}$, ${\text{AAL}}_{{\text{C}}\$}$, and ${\text{AAL}}_{{\text{U}}\$}$ is represented here as a proportion of $V_{{\text{R}}}$ (Eq. 18):

18 $${\text{AAL}}_{{\text{T}}\$} = \left( {{\text{AAL}}_{{\text{B}}} {\text{ + AAL}}_{{\text{C}}} { + }{\frac{{{\text{AAL}}_{{{\text{Use}}}} }}{{84 }}}} \right) \times V_{{\text{R}}} {.}$$

The effect of adding additional elevation above the BFE is considered by adding one foot incrementally up to four feet, and the relative reduction in flood risk (${\text{AAL}}_{{{\text{Red}}}}$) as result of home elevating is calculated here by using Eq. (19).

19 $${\text{AAL}}_{{{\text{Red}}}} \% = {\frac{{{\text{AAL}}_{{{\text{BFE}}}} {{ {-} AAL}}_{{{{{\text {BFE}}^{\prime}}}}} }}{{{\text{AAL}}_{{{\text{BFE}}}}} }}{.}$$

Here, ${\text{AAL}}_{{{\text{BFE}}}}$ is the AAL at BFE and ${\text{AAL}}_{{{{{\text {BFE}}^{\prime}}}}} { }$ represents the AAL at an additional elevation above BFE.

### Data Processing

The flood risk assessment in this study is conducted using the algorithm developed by Al Assi et al. (2023a) to perform flood risk assessment for every possible combination of $u$ and $a$. The framework is chosen due to its unique approach to assessing flood risk, as it divides the AAL into separate components for the building, contents, and use, by owner/occupant type, leading to a more detailed analysis of flood risk.

In addition, the framework includes a calculation of the AAL reduction associated with increasing the elevation above the BFE. This computation is crucial for understanding the extent of risk reduction achievable through home elevation. To perform this analysis, the algorithm requires specific information, including the number of stories, presence of a basement, living area ($A$), unit cost per square footage ($C_{{\text{R}}}$), and BFE.

The algorithm incorporates the building and contents loss DDFs from the United States Army Corps of Engineers (USACE 2000), taking into account home attributes such as the number of stories and the presence of a basement. Additionally, the algorithm includes the DDFs for use loss from FEMA (2013), based on the type of home and owner/occupant. For each flooding case, the algorithm inputs the valid combination of flood parameters ($u$ and $a$) along with each home attribute (e.g., one story with or without a basement).

### Results Description

The test is utilized to evaluate whether each data set follows a normal distribution. In cases of normally distributed (*α *< 0.05) data, the results are presented in terms of the average and standard deviation values. For data sets that are not normally distributed (*α* > 0.05), the results are expressed as quartiles or percentiles. Each data set is divided into quartiles, including the minimum and maximum values. The three quartiles, namely, Q1, Q2, and Q3, correspond to the 25th, 50th, and 75th percentiles, respectively. This indicates that 25%, 50%, and 75% of the data set falls below the defined value at each quartile.

### Case Study Validation

To validate the flood risk assessment generated using synthetic parameters, available flood depth data at 10-, 50-, 100-, and 500-year return periods for locations in California, Colorado, Michigan, New Hampshire, New Jersey, and Oregon, representing a variety of flood characteristics, are used to generate real flood parameters and characterize the flood risk. For each data set in each location, the real flood parameters are generated based on the flood depth data availability. The data are then divided into three cases based on data availability in the locations flooded at return periods exceeding: 1.58 years (i.e., all flood depth data available; Case 1), 10 years (i.e., data available for 50-, 100-, and 500-year return periods; Case 2), and 50 years (i.e., data available for 100- and 500-year return periods; Case 4). It should be noted that Case 3 is omitted from this analysis due to data unavailability. The flood case, 100-year flood depth, and hypothetical home attributes are used to generate a variety of scenarios. These combinations are then compared with the flood risk estimates generated using synthetic parameters.

## Results

### Synthetic Flood Parameters

To determine the $u$ parameter range and the combinations that satisfy A Zone conditions, the ratio of flood parameters (Eq. 11) and the upper limits of the $a$ parameter are determined. Given that the greatest value of the $a$ parameter is 4.60, the range of $u$ is found to be between – 21.16 and 0, as shown in Eq. (20):

20 $$- \;21.16 \le u < 0.$$

A total of 4966, 2540, 1510, and 755 $u$ and $a$ combinations fall within the possible ranges according to the $u$ to $a$ ratio for the A Zone for flooding in Cases 1, 2, 3, and 4 (Eq. 11), respectively. The distribution of $u$ and $a$ values resulting from these combinations is analyzed and is found to be non-normal, as indicated by the normality test with $p$-value > 0.005, suggesting that the data set for $a$ and $u$ parameters is not normally distributed. Therefore, the results are presented as 25, 50, and 75 percentiles along with the minimum and maximum values (Table 1).

**Table 1:** Descriptive statistics of synthetic flood parameters in the A Zone

Flooding case	Flood parameter	Minimum	25th	50th	75th	Maximum
Case 1	$u$	− 21.06	− 10.66	− 6.26	− 2.86	− 0.06
	$a$	0.10	2.30	3.30	4.00	4.60
Case 2	$u$	− 21.06	− 13.76	− 10.56	− 7.46	− 0.26
	$a$	0.10	2.30	3.30	4.00	4.60
Case 3	$u$	− 21.06	− 15.46	− 12.61	− 8.91	− 0.36
	$a$	0.10	2.30	3.30	4.00	4.60
Case 4	$u$	− 21.06	− 17.06	− 13.96	− 9.86	− 0.46
	$a$	0.10	2.30	3.30	4.00	4.60

**Fig. 1:**
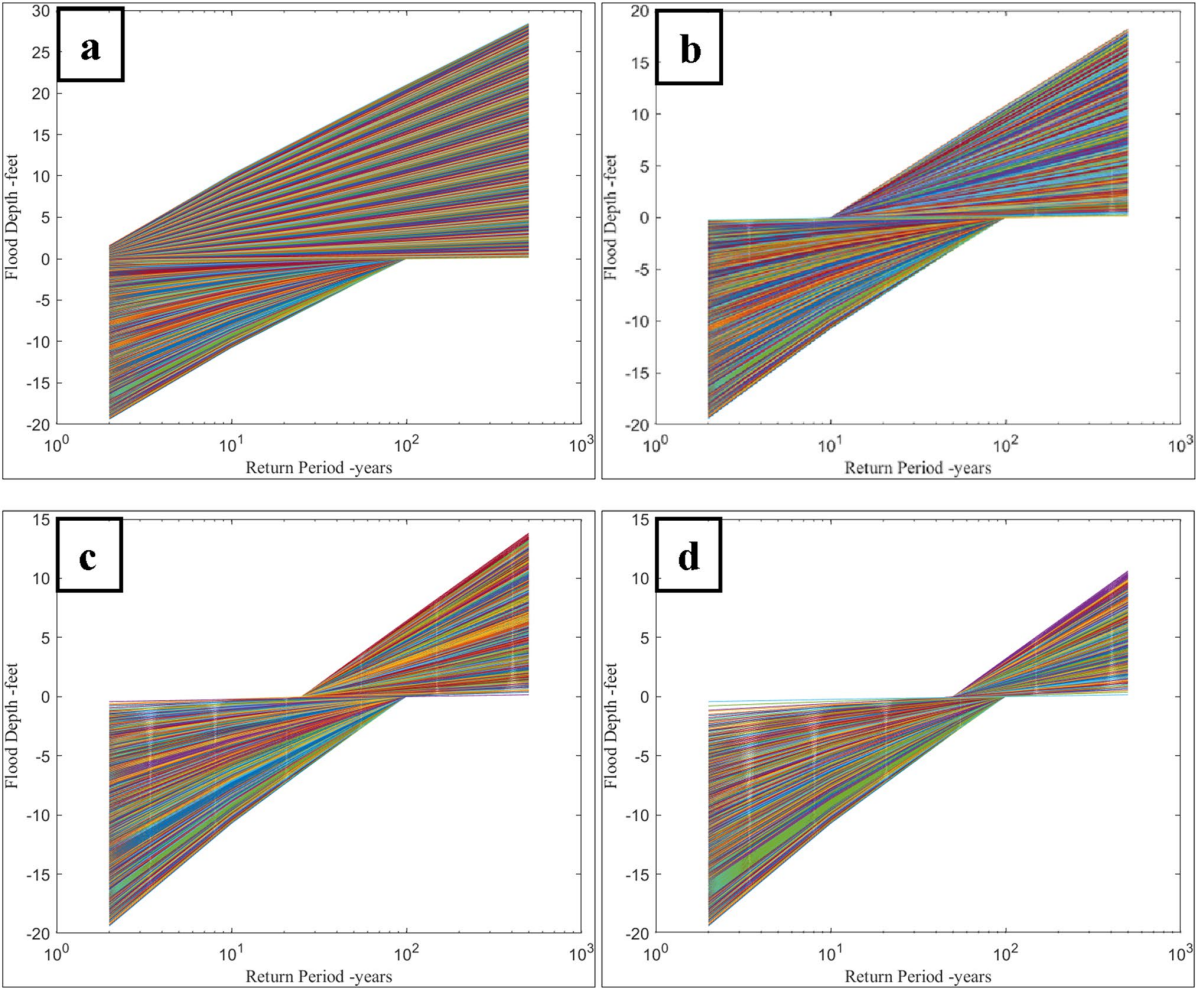
Relationship between flood depth and return period for synthetic data at four flooding cases in the A Zone: **a** Case 1, locations flooded at return periods exceeding 1.58 years, **b** Case 2, locations flooded at return periods exceeding 10 years, **c** Case 3, locations flooded at return periods exceeding 25 years, **d** Case 4, locations flooded at return periods exceeding 50 years

The valid $u$ and $a$ combinations are included in the computation of the flood depth–return period relationships generated for each flooding case, as shown in Fig. 1. All possible combinations for flood parameters generate a positive flood depth for a 100-year return period and longer, meeting the A Zone definition. The relationship derived from Case 1 serves as a baseline, yielding the most conservative estimate of flood risk in the A Zone. When flood depth data for return periods other than 100 years are unavailable, Case 1 data would be appropriate, yet would tend to overestimate flood risk. The remaining cases demonstrate how this relationship changes when additional flood data are available, represented in flood depth data at return periods smaller than 100 years.

The detailed descriptive statistics for 100-year flood depths for each flooding case are shown in Table 2. The minimum 100-year flood depth for all cases is 1.49*$10^{ - 5}$ feet while the maximum value ranges from 3.2 to 21.10 feet.

**Table 2:** Descriptive statistics of 100-year flood depth for each flooding case in A Zone

Flooding case	Minimum (feet)	25th (feet)	50th (feet)	75th (feet)	Maximum (feet)
Case 1	1.49*$10^{ - 5}$	2.86	6.26	10.68	21.10
Case 2	1.49*$10^{ - 5}$	1.46	3.20	5.47	10.80
Case 3	1.49*$10^{ - 5}$	0.88	1.92	3.26	6.40
Case 4	1.49*$10^{ - 5}$	0.44	0.96	1.64	3.20

### Annual Flood Risk and Risk Reduction

Annual flood risk as a proportion of $V_{{\text{R}}}$ at the lowest floor elevation at BFE and at each additional elevations above BFE is calculated for the 4,966, 2,540, 1,510, and 755 combinations of valid flood parameters in the A Zone (Tables 3, 4, 5 and 6). The results for each flooding case are categorized by 100-year flood depth and home attributes (e.g., one story or two-plus story, each without or with a basement). As the AAL results are not normally distributed, the ratios of total AAL to $V_{{\text{R}}}$ (i.e., ${\text{AAL}}_{{T/V_{{\text{R}}} }}$) are presented in terms of percentiles.

**Table 3:** Descriptive statistics of average annual loss as a proportion of $V_{R}$ (i.e., ${\text{AAL}}_{{T/V_{{\text{R}}} }}$) categorized by 100-year flood depth and AAL quartile for single-family home in the A Zone, using synthetic data, for Case 1 (i.e., flooding at return periods exceeding 1.58 years)

	Total average annual loss as a proportion of $V_{{\text{R}}}$ (i.e., ${\text{AAL}}_{{T/V_{{\text{R}}} }}$) × 10^–3^									
	One story, no basement					One story + basement				
100-year flood depth (feet)	Min	25th	50th	75th	Max	Min	25th	50th	75th	Max
< 1	3.60	4.94	6.33	7.38	8.24	6.17	7.10	8.21	9.09	9.80
1–2	3.77	5.19	6.43	7.47	8.24	6.28	7.29	8.30	9.16	9.80
2–3	4.03	5.42	6.53	7.47	8.24	6.45	7.48	8.38	9.16	9.80
3–4	4.42	5.66	6.68	7.55	8.24	6.72	7.66	8.47	9.23	9.80
4–5	4.68	5.89	6.83	7.63	8.24	6.91	7.85	8.63	9.27	9.80
5–6	4.94	6.03	6.93	7.63	8.24	7.10	7.94	8.71	9.30	9.80
6–7	5.31	6.22	7.02	7.71	8.24	7.38	8.12	8.79	9.37	9.80

	Two-plus story, no basement					Two-plus story + basement				
	Min	25th	50th	75th	Max	Min	25th	50th	75th	Max
< 1	2.74	3.65	4.72	5.61	6.39	4.93	5.57	6.46	7.29	8.05
1–2	2.85	3.83	4.80	5.69	6.39	5.00	5.71	6.54	7.36	8.05
2–3	3.02	4.01	4.89	5.69	6.39	5.11	5.85	6.62	7.36	8.05
3–4	3.29	4.19	5.01	5.76	6.39	5.30	6.00	6.69	7.43	8.05
4–5	3.47	4.39	5.14	5.84	6.39	5.30	6.00	6.69	7.43	8.05
5–6	3.65	4.49	5.22	5.84	6.39	5.43	6.16	6.84	7.51	8.05
6–7	3.92	4.63	5.30	5.91	6.39	5.57	6.23	6.92	7.51	8.05

Results reveal that the median AAL falls between 0.47 and 0.98 percent of $V_{{\text{R}}}$ for a single-family home located in A Zone considering all flooding cases. These findings highlight the variability of AAL among different home types, primarily influenced by the unique DDFs associated with each type. Specifically, homes with basements show higher AAL compared to those without, and one-story home experiences greater AAL than their two-story. Not surprisingly, flood depth is the important factor involved in flood risk, with greater depth causing more damage.

Table 3 provides a broad estimation of flood risk in the A Zone, particularly when flood depth data for return periods other than 100 years are unavailable. The other cases presented in Tables 4, 5 and 6 showcase the refined flood risk assessment achieved with additional flood data. The results show that there are slight variations between Case 1 and the remaining cases for the same home type and a 100-year flood depth.

**Table 4:** As in Table 3, for Case 2 (i.e., flooding at return periods exceeding 10 years)

	Total average annual loss as a proportion of $V_{{\text{R}}}$ (i.e., ${\text{AAL}}_{{T/V_{{\text{R}}} }}$) × 10^–3^									
	One story, no basement					One story + basement				
100-year flood depth (feet)	Min	25th	50th	75th	Max	Min	25th	50th	75th	Max
< 1	3.60	5.06	6.33	7.38	8.24	6.17	7.19	8.21	9.09	9.80
1–2	4.03	5.43	6.53	7.47	8.24	6.45	7.48	8.42	9.20	9.80
2–3	4.55	5.89	6.83	7.59	8.24	6.81	7.85	8.63	9.30	9.80
3–4	5.06	6.22	7.02	7.71	8.24	7.19	8.12	8.79	9.37	9.80
4–5	5.66	6.53	7.20	7.79	8.24	7.66	8.38	8.94	9.43	9.80
5–6	6.11	6.83	7.38	7.87	8.24	8.03	8.63	9.10	9.50	9.80
6–7	6.53	7.29	7.63	7.95	8.24	8.38	8.87	9.23	9.56	9.80

	Two-plus story, no basement					Two-plus story + basement				
	Min	25th	50th	75th	Max	Min	25th	50th	75th	Max
< 1	2.74	3.74	4.72	5.61	6.39	4.93	5.64	6.46	7.30	8.05
1–2	3.02	4.01	4.89	5.69	6.39	5.11	5.85	6.65	7.40	8.05
2–3	3.38	4.37	5.14	5.80	6.39	5.36	6.16	6.84	7.51	8.05
3–4	3.74	4.63	5.30	5.91	6.39	5.64	6.39	7.00	7.58	8.05
4–5	4.19	4.89	5.46	5.98	6.39	6.00	6.62	7.14	7.65	8.05
5–6	4.54	5.14	5.61	6.05	6.39	6.31	6.84	7.30	7.71	8.05
6–7	4.89	5.38	5.76	6.12	6.39	6.62	7.07	7.43	7.78	8.05

**Table 5:** As in Table 3, for Case 3 (i.e., flooding at return periods exceeding 25 years)

	Total average annual loss as a proportion of $V_{{\text{R}}}$ (i.e., ${\text{AAL}}_{{T/V_{{\text{R}}} }}$) × 10^–3^									
	One story, no basement					One story + basement				
100-year flood depth (feet)	Min	25th	50th	75th	Max	Min	25th	50th	75th	Max
< 1	3.6	5.19	6.43	7.47	8.24	6.17	7.29	8.30	9.16	9.80
1–2	4.42	5.89	6.83	7.63	8.24	6.72	7.85	8.63	9.30	9.80
2–3	5.31	6.43	7.11	7.71	8.24	7.38	8.30	8.87	9.37	9.80
3–4	6.11	6.93	7.47	7.87	8.24	8.03	8.71	9.16	9.50	9.80
4–5	6.93	7.47	7.72	8.02	8.24	8.71	9.16	9.37	9.63	9.80
5–6	7.47	7.87	8.02	8.17	8.24	9.16	9.50	9.63	9.74	9.80
6–7	8.02	8.17	8.21	8.24	8.24	9.63	9.75	9.78	9.80	9.80

	Two-plus story, no basement					Two-plus story + basement				
	Min	25th	50th	75th	Max	Min	25th	50th	75th	Max
< 1	2.74	3.83	4.80	5.69	6.39	4.93	5.71	6.54	7.36	8.05
1–2	3.29	4.37	5.14	5.84	6.39	5.30	6.16	6.84	7.51	8.05
2–3	3.92	4.80	5.38	5.91	6.39	5.78	6.54	7.07	7.58	8.05
3–4	4.54	5.22	5.69	6.05	6.39	6.31	6.92	7.36	7.71	8.05
4–5	5.22	5.69	5.91	6.19	6.39	6.92	7.36	7.58	7.85	8.05
5–6	5.76	6.05	6.19	6.33	6.39	7.36	7.71	7.85	7.98	8.05
6–7	6.19	6.33	6.36	6.39	6.39	7.85	7.98	7.98	8.05	8.05

**Table 6:** As in Table 3, for Case 4 (i.e., flooding at return periods exceeding 50 years)

	Total average annual loss as a proportion of $V_{{\text{R}}}$ (i.e., ${\text{AAL}}_{{T/V_{{\text{R}}} }}$) × 10^–3^									
	One story, no basement					One story + basement				
100-year flood depth (feet)	Min	25th	50th	75th	Max	Min	25th	50th	75th	Max
< 1	3.60	5.55	6.64	7.55	8.24	6.17	7.57	8.47	9.23	9.80
1–2	5.31	6.66	7.29	7.79	8.24	7.38	8.55	9.01	9.43	9.80
2–3	6.93	7.55	7.87	8.10	8.24	8.71	9.23	9.50	9.69	9.80
3–4	8.10	8.17	8.21	8.24	8.24	9.69	9.75	9.78	9.80	9.80

	Two-plus story, no basement					Two-plus story + basement				
	Min	25th	50th	75th	Max	Min	25th	50th	75th	Max
< 1	2.74	4.10	4.97	5.76	6.39	4.93	5.93	6.69	7.43	8.05
1–2	3.92	4.99	5.54	5.98	6.39	5.78	6.77	7.22	7.65	8.05
2–3	5.22	5.76	6.05	6.26	6.39	6.92	7.43	7.71	7.92	8.05
3–4	6.26	6.33	6.36	6.39	6.39	7.92	7.98	8.01	8.05	8.05

The relative reduction results in Table 7 show the percentage of reduction for minimum, maximum, and quartile annual risk values. The results demonstrate that the AAL reduction varies based on the 100-year flood depth range and total AAL quartiles. The maximum reduction is achieved in areas between the minimum and 25th percentile of AAL, while this reduction decreases in areas with greater total AAL and greater 100-year flood depths. The general principle is that freeboard results in meaningful risk reduction, particularly for areas with a 100-year flood depth of less than 1 foot and where AAL is minimal; in such places, any amount of freeboard results in greater than 99% reduction of annual flood risk. For instance, for 100-year flood depth less than 1 foot, a 1-foot increase above BFE results in a risk reduction of more than 99% for the smallest observed flood (i.e., 3.60 × 10^–3^ of $V_{{\text{R}}}$), a 33% risk reduction for the median observed flood (i.e., i.e., 6.63 × 10^–3^ of $V_{{\text{R}}}$), and a 19% risk reduction for the greatest observed flood risk (i.e., 8.24 × 10^–3^ of $V_{{\text{R}}}$).

**Table 7:** Relative annual flood risk reduction ${\text{(AAL }}_{{{\text{Red}}}} {\% }$) with freeboard for a single-family home at each 100-year flood depth

		Relative annual flood risk reduction (%)				
100-year flood depth (feet)	First-floor height (feet)	Min	25th	50th	75th	Max
< 1	BFE + 1	> 99%	50%	33%	25%	19%
	BFE + 2	> 99%	76%	55%	43%	35%
	BFE + 3	> 99%	88%	70%	57%	48%
	BFE + 4	> 99%	94%	80%	67%	58%
1–2	BFE + 1	76%	42%	30%	23%	19%
	BFE + 2	91%	65%	50%	41%	35%
	BFE + 3	96%	79%	65%	55%	48%
	BFE + 4	98%	87%	75%	65%	58%
2–3	BFE + 1	66%	38%	28%	23%	19%
	BFE + 2	84%	62%	49%	41%	35%
	BFE + 3	91%	75%	63%	54%	48%
	BFE + 4	95%	84%	74%	65%	58%
3–4	BFE + 1	46%	32%	26%	22%	20%
	BFE + 2	67%	53%	44%	39%	35%
	BFE + 3	78%	67%	58%	52%	48%
	BFE + 4	85%	76%	69%	63%	58%

### Case Study Validation

**Fig. 2:**
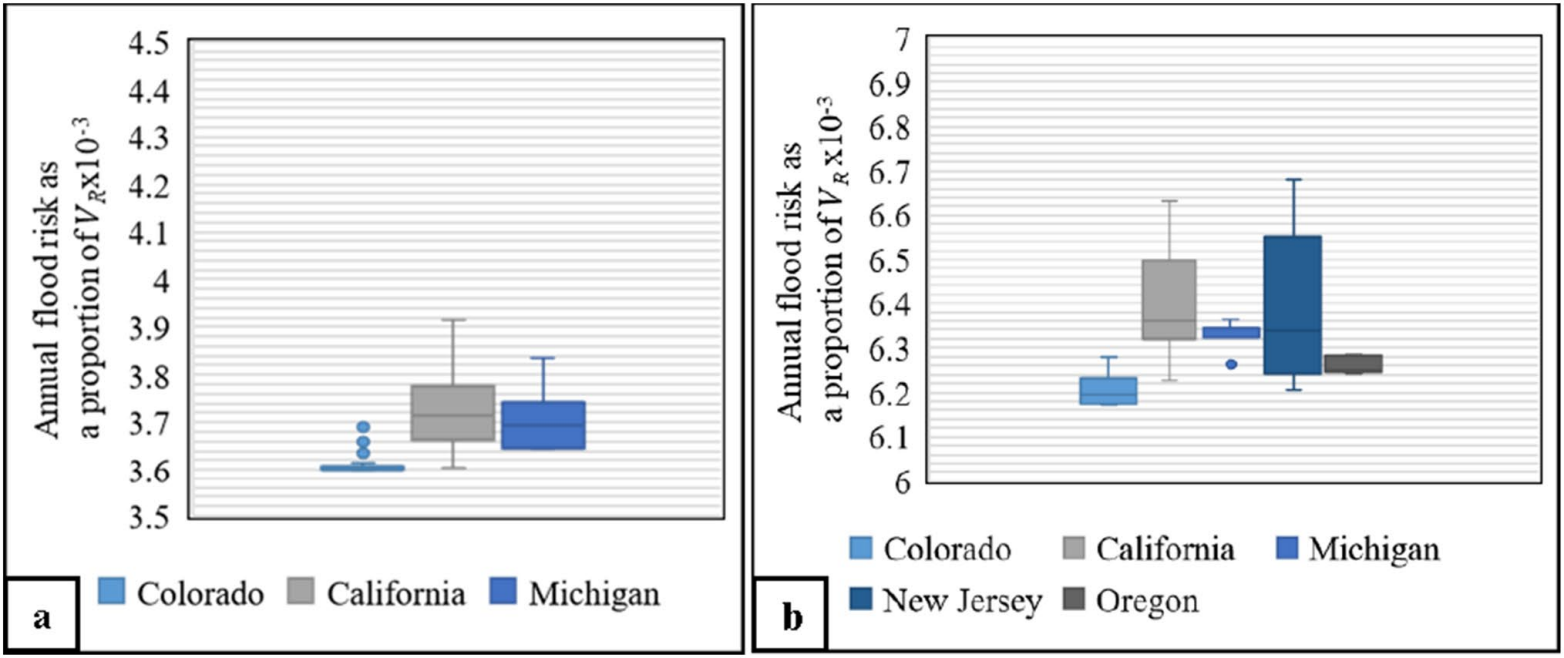
Flood risk analysis for single-family homes in Case 1 (i.e., flooded at return periods exceeding 1.58 years). The analysis considers two scenarios: **a** one-story homes without a basement, having a 100-year flood depth of less than one foot, and **b** one-story homes with a basement, having a 100-year flood depth from 1 to 2 feet

**Fig. 3:**
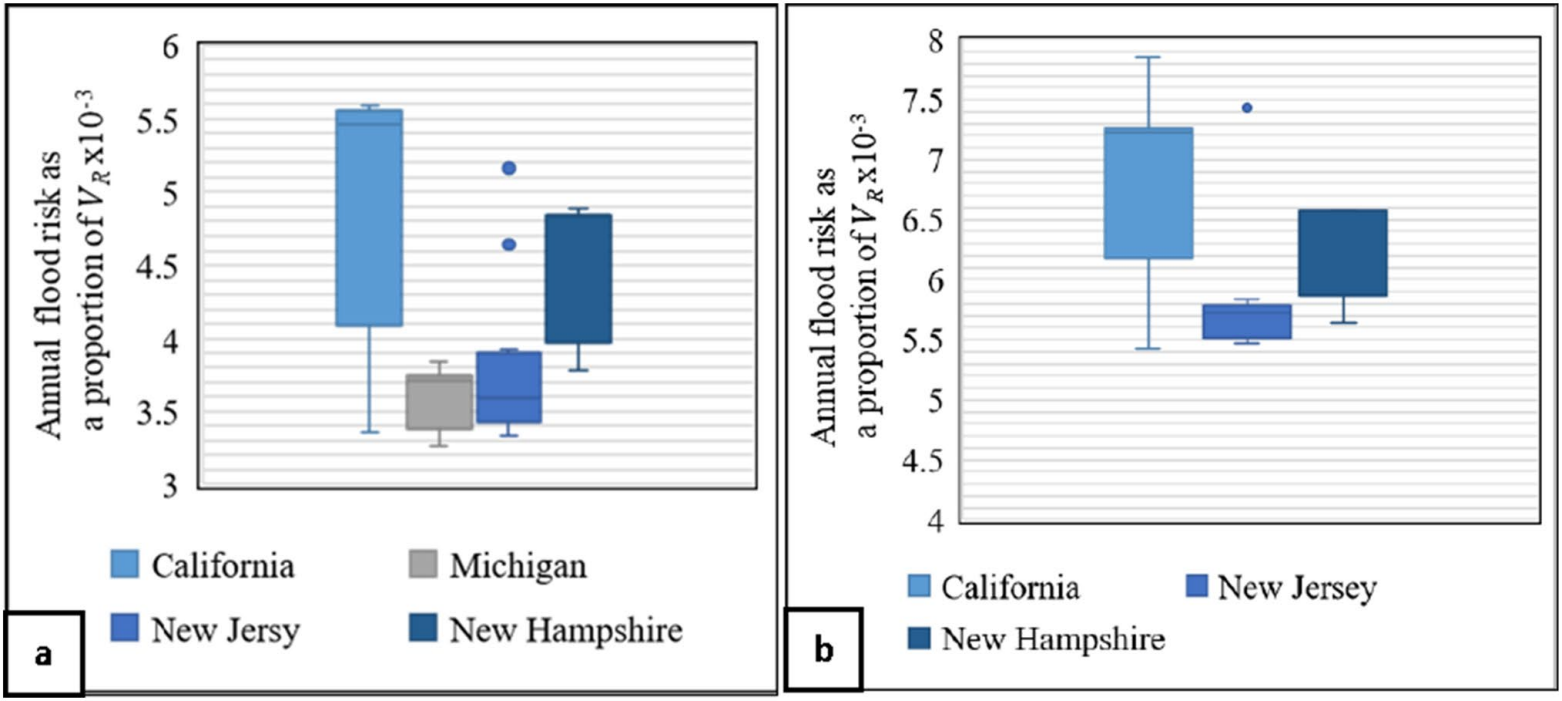
Flood risk analysis for single-family homes in Case 2 (i.e., flooded at return periods exceeding 10 years). The analysis considers two scenarios: **a** two-story homes without a basement, having a 100-year flood depth ranging from 2 to 3 feet, and **b** two-story homes with a basement, having a 100-year flood depth ranging from 3 to 4 feet

**Fig. 4:**
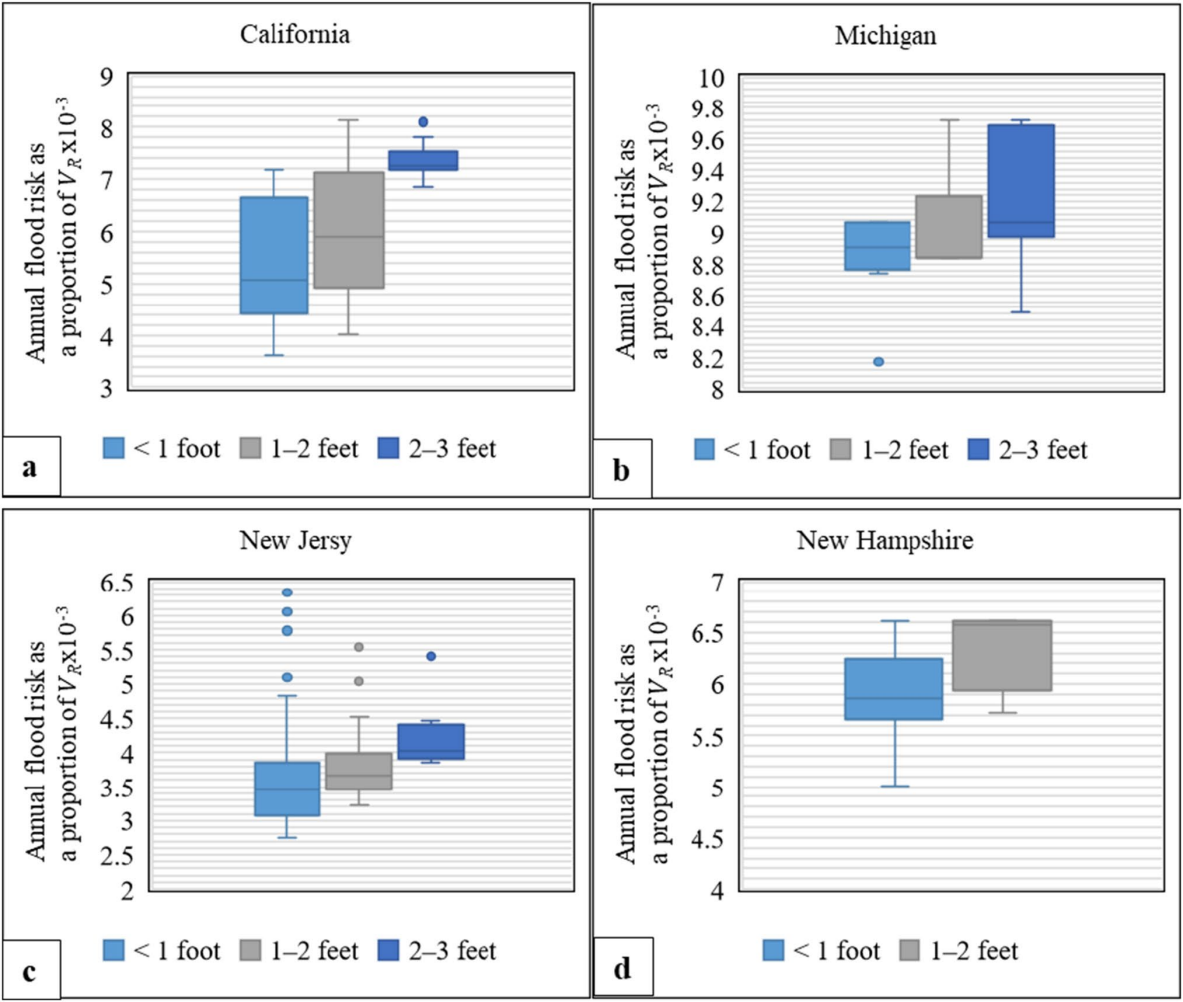
Flood risk analysis for single-family homes in Case 4 (i.e., flooded at return periods exceeding 50 years). The analysis considers four locations with various home attributes by 100-year flood depth: **a** one-story homes without a basement in California, **b** one-story homes with basement in Michigan, **c** two-story homes without a basement in New Jersey, and d) two-story homes with basement in New Hampshire

The descriptive statistical results of the annual flood risk as a proportion of $V_{R}$ are presented in the form of boxplots in Figs. 2, 3, 4. These boxplots show the minimum, median, and maximum values, as well as any outlier points beyond the range, providing a comprehensive analysis of the flood risk for various scenarios in California, Colorado, Michigan, New Hampshire, New Jersey, and Oregon. These scenarios are categorized based on the flooding case, 100-year flood depth, and home attributes (single-family homes with one story or two-plus story, each without and including a basement). The results show that the flood risk in these locations is within the range of synthetic results. For example, results for Case 1 (flooded at return periods exceeding 1.58 years), as shown in Fig. 2a, reveal that the flood risk for single-family homes with one story and no basement located in Colorado, California, and Michigan with 100-year flood depth less than 1.0 feet falls between the minimum and 25th percentiles of the synthetic results (as per Table 3). Additionally, Case 1 one-story homes located in Colorado, California, Michigan, New Jersey, and Oregon, with a basement and a 100-year flood depth between 1 and 2 feet (Fig. 2b) have a flood between the minimum and 25th percentiles of synthetic data (as per Table 3).

For Case 2 (flooded at return periods exceeding 10 years) with a 100-year flood depth range of 2–3 feet, the lower bound of flood risk results for single-family homes with two stories and no basement is within the range of the minimum synthetic flood results, while the upper bound of flood risk results is within the range of the 75th, 25th, and the 50th percentiles of the synthetic results in California, Michigan, and New Jersey/New Hampshire, respectively (Fig. 3a; Table 4). Additionally, for the same flooding case with a 100-year flood depth range of 3–4 feet for single-family homes with two stories and a basement, the lower bound of flood risk results is within the range of the minimum synthetic flood results, while the upper bound of flood risk results is within the range of the maximum, and 75th and 50th percentiles of the synthetic results in California, New Jersey, and New Hampshire, respectively (Fig. 3b; Table 4). Finally, Fig. 4 presents the results of Case 4 in multiple locations and for various 100-year flood depths. These results show that the flood risk for each location falls within the range of the synthetic data, based on the same category (Table 6).

**Fig. 5:**
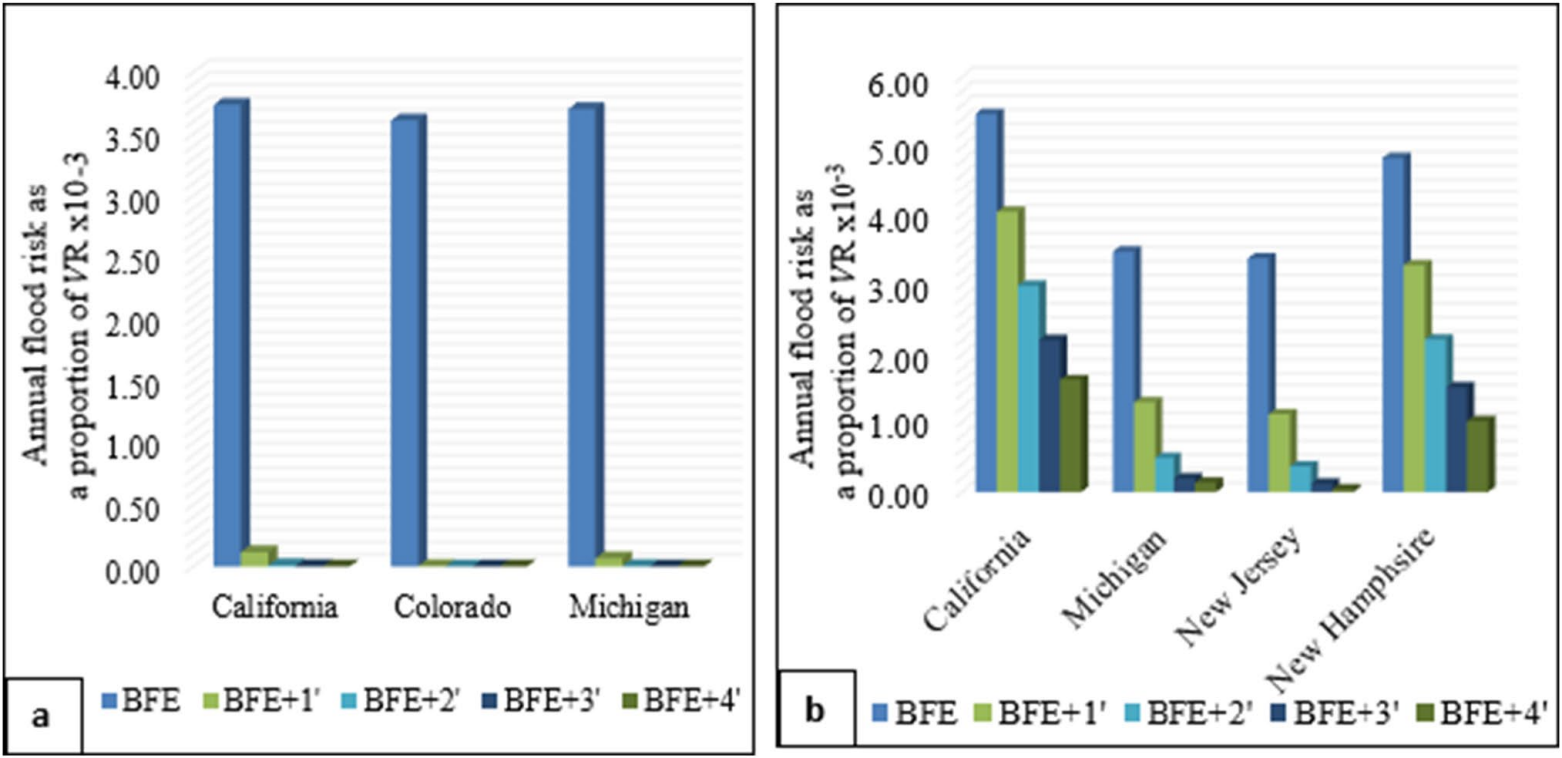
Median flood risk by each increment above BFE. The analysis considers two scenarios: **a** the first flooding case for one-story homes without a basement and a 100-year flood depth of less than one foot, and **b** the second flooding case for two-story homes with a basement with a 100-year flood depth ranging from 2 to 3 feet

The results for flood risk reduction through home elevation are presented in Fig. 5, which shows the effect of elevation on one-story homes located in Colorado, California, and Michigan for a 100-year flood depth of less than one foot (Fig. 5a). The analysis suggests that the median flood risk for homes with a 100-year flood depth of less than one foot is reduced by more than 99% when elevating the lowest floor by one foot above the BFE. The median flood risk is within the range of the minimum synthetic flood risk results (Fig. 2a; Table 3), resulting in a reduction that is consistent with the findings presented in Table 7.

Additionally, this study also analyzed homes located in California, Michigan, New Jersey, and New Hampshire with a 100-year flood depth range of 2–3 feet (Fig. 5b). Results show that elevating homes with a 100-year flood depth range of 2–3 feet by one foot above BFE reduced the median flood risk by 27%, 63%, 67%, and 33% in California, Michigan, New Jersey, and New Hampshire, respectively (Fig. 5b). These findings are consistent with Table 7 results as the median flood risk is within the 50th and 75th percentiles range of synthetic flood results in California, while it falls within the minimum range of synthetic flood results in Michigan and New Jersey, and the 25th percentile range of synthetic flood results in New Hampshire (Fig. 3a, Table 4).

## Discussion

The determination of synthetic flood parameters ($u$ and $a$; Table 1) for the A Zone yields valuable insights into the interplay between flood depth and return period, as depicted in Fig. 1. The findings in Fig. 1 offer valuable insights for informing flood risk management and decision-making processes by providing a comprehensive understanding of the range of expected flood depths at various return periods and enabling the determination of expected flood depths for various return periods along each line.

In FEMA flood zone designations (FEMA 2024), zones such as A, AE, AR, and A99 lack specific flood depth definitions for the 1% annual chance of flooding. In contrast, Zones AH and AO specify a range between 1 and 3 feet for this criterion. This study fills a notable gap by elucidating the 100-year flood depth in areas designated with the letter “A” (e.g., A Zone), showing a median depth exceeding 6 feet (Table 2). The observed variation in flood depth emphasizes the need for comprehensive assessment within FEMA flood zones. While certain zones provide clear depth ranges for a 1% annual chance of flooding, the A Zone, without such specificity, requires incorporating updated data into flood zone designations to better reflect the actual flood depth value.

Notably, this research differs from existing studies and tools (Armal et al. 2020, FEMA’s National Risk Index (NRI; Zuzak et al. 2022, 2023), Hazardaware 2024), by estimating AAL for single-family homes in the A Zone at the individual level, even in the absence of flood depth data. Moreover, this individualized assessment technique overcomes challenges associated with fluctuating asset values over time by providing the total annual flood risk (building, contents, and use) for single-family homes in the A Zone proportionally to $V_{R}$. It is anticipated that calculating results in this format will enhance applicability of the results over time in the A Zone with the actionable outcome of increasing awareness of the benefits of applying mitigation actions. Furthermore, the incorporation of loss of use improves upon previous studies as most residential flood risk assessment studies have focused only on building and contents risk to homeowners. However, loss of use is only one component of indirect loss. Future research should focus on further consideration of the assessment of indirect and intangible losses, which are considered critical metrics in assessing the multifaceted impacts of floods on residents.

The results of this research add to the important information that must be conveyed to decision-makers. However, the significance of these findings provides insight for individual homeowners who interpret the impact of flood risk at the individual level in intangible dollar values. This becomes particularly important when considering the construction at the minimum height, approximately equal to BFE, the national standard used by the NFIP and all federal agencies (FEMA 2005), or incorporating additional elevation above BFE (ASCE 2014).

The results demonstrate that, in all flooding cases, the AAL ranges from 0.3 to 1 percent of $$V_{{\text{R}}}$$ for single-family homes built at FFE equal to BFE, the minimum elevation, with median values falling between 0.47 and 0.98 percent of $$V_{{\text{R}}}$$ (Tables 3–6). These findings provide insight into the value of AAL in the A Zone. To illustrate, considering a scenario where the building replacement cost is $300,000, the annual flood risk ranges from $900 to $2,940 for all home attributes and 100-year flood depths. When these parameters are known, the range of AAL is refined. For example, for a one-story home with a basement with a 100-year flood depth between 2 and 3 feet considering flooding Case 3, the relative AAL range is 0.74–0.98 percent (Table 5), resulting in AAL between $2220 and $2940.

The result from this research emphasizes the importance of conducting a comprehensive flood risk assessment and implementing effective mitigation measurements not just in the A Zone but also in the shaded X Zone. By drawing a comparison, the shaded X Zone shows a median AAL range of 0.10–0.78 percent of replacement cost value, as reported by Al Assi et al. (2023c). For instance, for a home valued at $300,000, this translates to an AAL ranging between $300 and $2340. This indicates a high flood risk in this area despite its exclusion from the SFHA.

The presentation of results as quartiles shows variations in AAL at equivalent 100-year flood depths and home attributes, indicating factors beyond the 100-year floodplain affect AAL. This observation aligns with Rahim et al. (2022) findings, suggesting that BFE values have no impact on AAL calculations considering that buildings in SFHA typically adhere to FEMA’s minimum elevation requirements, effectively built the FFE at BFE. These results prompt a reconsideration of the focus on the 100-year flood as the controller parameter for defining SFHA and freeboard requirements, suggesting that future research should focus on other factors that influence AAL beyond a 1% annual chance flood.

Increasing the FFE above the BFE substantially decreased the flood risk in the A Zone, although the flood risk reduction (Table 7) depends on the 100-year flood depth and the flood risk range (Tables 3–6). The analysis demonstrates that adding one to four feet of additional elevation above the BFE results in a median AAL reduction of 30–75%, as shown in Table 7. Notably, areas falling within the minimum and 25th percentile flood risk range experience over 90% reduction in AAL with just one foot above BFE. These findings align with Al Assi et al. (2023a), who found that adding one foot above BFE reduces flood risk by 90% in a study area in Jefferson Parish, Louisiana.

This research underscores the caveat that the minimum elevation requirement of 1.0 feet above BFE for residential structures in the U.S.A. as stated by ASCE (2014) may not apply to all residential buildings located in the A Zone. Instead, a more comprehensive flood risk analysis is prudent when determining the appropriate elevation to minimize flood risk (Mostafiz et al. 2023). The decision-making may be implemented at the community scale to the design flood elevation (DFE). ASCE (2014) defines the DFE as “elevation of the flood associated with the greater of the following two areas: (1) area within a floodplain subject to a 1% or greater chance of flooding in any year, or (2) area designated as a flood hazard area on a community’s flood hazard map or otherwise legally designated, including wave height, relative to the datum specified on the community’s flood hazard map.”

The AALs for the case study subsets of California, Colorado, Michigan, New Hampshire, New Jersey, and Oregon generated by available flood depth data at 10-, 50-, 100-, and 500-year return period are within the range of AAL results of the synthetic results, considering the various ranges of 100-year flood depths and home attributes. The flood risk for homes located in California, Colorado, and Michigan with a 100-year flood depth of less than two feet (Fig. 2a) is between the minimum and 25th percentile, indicating that adding just one foot above the BFE reduces the flood risk substantially (Table 7 and Fig. 5a). Areas within California, Colorado, Michigan, New Hampshire, and New Jersey with 100-year flood depth exceeding two feet have a flood risk ranging between the minimum and 75th percentile (Fig. 3a), suggesting that elevating homes by two or three feet above the BFE is necessary to decrease the flood risk by over 50% (Table 7 and Fig. 5b).

While both techniques lead to similar results, additional validation to confirm the range of synthetic flood parameters for various areas will further support their use in similar risk assessments and flood risk investigations in the V Zone and in areas where the $a$ parameter exceeds 4.6. Also, it is important to note that these results depend on many parameters, particularly the selection of DDF. Further analyses conducted within this research show that utilizing Nofal et al.’s (2020) DDF for single-family homes, including building and contents loss only, yields median AAL ranging from 0.05 to 0.5 percent of $V_{{\text{R}}}$. Therefore, future research is needed to focus on refining and enhancing the accuracy of DDFs.

## Conclusion

This paper implements a synthetic analytical approach to comprehensively characterize flood risk in the A Zone. By defining four distinct flooding cases (i.e., location flooded at return periods exceeding 1.58-, 10-, 25-, and 50-year return period events), a library of synthetic flood parameters is generated and used to assess flood risk for hypothetical single-family homes in the U.S.A. (i.e., one vs. two-plus stories, with vs. without basement) located in the A Zone. This study also explores the relative reduction in flood risk achieved with each additional FFE increment (i.e., one to four feet) above the BFE. Actual flood parameters are derived from available flood depth data and relative AAL is compared with synthetic values for case studies in California, Colorado, Michigan, New Hampshire, New Jersey, and Oregon.

The major findings are as follows:

- The synthetic flood parameter approach generates multiple combinations of flood parameters for each flooding case, involving a wide range of 100-year flood depths.
- The flood depth–return period relationship for each flooding case, including all synthetic flood parameter combinations, provides vital information about flood depth by return period.
- The AAL for all flooding cases, taking into account four types of single-family homes (one story and two-plus story, without basement and with basement) in the A Zone ranges from 0.3 to 1.0 percent of the replacement cost value when considering the analyzed synthetic data.
- The relative flood risk is reduced by more than 90% by adding one foot of freeboard if the annual flood risk falls between the minimum and 25th percentiles with a 100-year flood depth of less than two feet. For areas falling between the 75th percentile and maximum annual flood risk, three feet of freeboard is needed to reduce flood risk by 50%.
- The descriptive statistical results for flood parameters, risk, and risk reduction with additional freeboard enhance the understanding of flood risk and the benefits of elevating above the BFE.
- For case study areas in California, Colorado, Michigan, New Hampshire, New Jersey, and Oregon, AAL values calculated from flood depth grids fall within the range of those computed from synthetic flood parameters.

The results provide an important first step for predicting and enhancing community understanding of flood risk even in the absence of flood depth data. The approach not only fills a critical gap by estimating the range of 100-year flood depth in A Zone but also provides insights into flood risk as a proportion of replacement cost value and in quartiles. The incorporation of elevation strategy enhances awareness, emphasizing the importance of adapting mitigation measures in flood-prone areas. Furthermore, providing specific recommended elevation for each location contributes to enhanced individual- and community-level awareness. These features make the approach widely applicable as populations continue to increase in areas in which the flood risk is unknown due to absent or outdated data.

This research provides valuable insights beyond the academic research. The practical implications of this research support homeowners, decision-makers, and developers alike. Its contributions include enhanced homeowner awareness, helping decision-makers in formulating effective policies, and enhancing requirements for protection measures. It guides the formulation of insurance policies and contributes to enhancing the protection measures requirements.

Although this study provides a crucial first step for predicting and enhancing community understanding of the flood risk in the A Zone, several precautions need to be considered. First, this study results will differ from those suggested here in areas where the $a$ parameter exceeds 4.60. Future research should expand to include such areas. Despite the fact that the A Zone is part of the SFHA, the V Zone coastal area is exposed to higher flood risk; thus, future research should expand its scope to include the V Zone, providing a more comprehensive assessment of flood risk.

While the flood building and content loss functions used in this research are among the most acceptable flood loss functions, further investigation is needed to develop site-specific flood depth vs. damage functions. Also, exploring the potential use of building-specific and component-based functions is warranted.

It is essential to acknowledge the study is limited to single-family homes, suggesting the potential expansion to multi-story buildings. In the present research, loss of use is considered, representing a significant advancement compared to previous analyses. However, it is important to recognize that this study did not take into account other losses avoided, such as function losses, indirect, and intangible. Future research should include such components of the losses. Despite these cautions, this research contributes to the mitigation of the loss experienced inside the SFHA and to improved awareness of the magnitude of flood risk in this region and the benefit of applying elevation strategies.

### Supplementary Information

Below is the link to the electronic supplementary material.

Supplementary file1 (DOCX 36 kb)
