# Supplementary material for: Theoretical Boundaries of Annual Flood Risk for Single-Family Homes Within the 100-Year Floodplain
Source: Int J Environ Res. 2024 Mar 15;18(2):29. doi: 10.1007/s41742-024-00577-7 (PMC10943154; doi:10.1007/s41742-024-00577-7)
Supplement: Supplementary file 1 — Supplementary file1 (DOCX 36 kb) [file 41742_2024_577_MOESM1_ESM.docx]

# Supplementary Materials

**Supplementary Table.** Variables: Description, Data Sources, and Scale of Measurement

|  | Variable | Abbreviation | Description | Data source | Scale of measurement |
| --- | --- | --- | --- | --- | --- |
| Flood Zone | Base flood elevation | BFE | The national standard used by the NFIP and all federal agencies and it is approximately equal to the 100-year flood elevation | FEMA Flood Insurance Rate Maps (FIRMs) | Feet |
|  | Special Flood Hazard Area | SFHA | The area inundated by the flood event has a 1% chance of being equaled or exceeded in any given year. | N/A | N/A |
|  | Zone A | Zone A | Areas are subject to inundation by a 1% annual chance of flooding where wave action does not occur or waves are less than 3 feet high. | N/A | N/A |
|  | Zone V | Zone V | The portion of the coastal SFHA where base flood wave heights  are 3 feet or greater, or where other damaging base flood wave effects have been identified, or  where the primary frontal dune has been identified. | N/A | N/A |
|  | Zone X  (Shaded) | Zone X (Shaded) | Areas outside the SFHA are subject to inundation by the flood that has a 0.2-percent chance of being equaled or exceeded during any given year, often referred to as a 500-year flood. | N/A | N/A |
|  | Zone X (Unshaded) | Zone X (Unshaded) | Areas outside the Special Flood Hazard Area where the annual  probability of flooding is less than 0.2 percent. | N/A | N/A |
| Flood Data | Annual exceedance probability | AEP ($P$) | The probability that a certain flood event will occur at least once in a given year | FEMA Flood Insurance Rate Maps (FIRMs) | Percentage |
|  | Annual probability of non-exceedance | $p$ | The complementary cumulative probability is the probability of a certain flood event not occurring in a given year | FEMA Flood Insurance Rate Maps (FIRMs) | Percentage |
|  | Flood return period | $T$ | The average time between the occurrence of a specific event at a given location. | N/A | Years |
|  | Location parameter for Gumbel distribution | $u$ | The intercept in the relationship between $d$ and the double natural logarithm of $P$ | Calculated | N/A |
|  | Scale parameter for Gumbel distribution | $a$ | The slope in the relationship between $d$ and the double natural logarithm of $P$ | Calculated | N/A |
|  | Depth-damage function | DDF | Characterize the relationship between the water depth in the structure and the percent of damage | USACE (2000) | Proportion of $V_{R}$(%) |
|  | Building loss | $L_{B}$ | Damage caused to the physical structure of the building, such as walls and roof | USACE (2000) | Proportion of $V_{R}$(%) |
|  | Contents loss | $L_{C}$ | Damage caused to the contents of the building, such as furniture. | USACE (2000) | Proportion of $V_{R}$(%) |
|  | Use loss | $L_{U}$ | The time required for the inhabitant to repair, clean up, and inspect the building | FEMA (2013) | Months |
|  | Flood depth in the structure | $dh$ | The height of the flood water inside a building or structure | Calculated | Feet |
|  | Flood depth above the ground | $d$ | The height of the flood water above the ground level | Risk Mapping, Assessment and Planning (RiskMAP) program | Feet |
|  | Rent loss | $R_{l}$ | Monthly rent incurred by the homeowner | Calculated | U.S dollars |
| Home attributes | Home area | $A$ | The total floor area of the home | Assumed based on the statistical data from | Square feet |
|  | Unit price cost | $C_{R}$ | The cost to repair one square foot of damage to the home | Assumed based on the statistical data from | U.S dollars per square feet |
|  | Replacement cost value | $V_{R}$ | The cost to rebuild a damaged property | Calculated | U.S dollars |
|  | First-floor elevation | FFE | The height of the first floor above ground level | Assumed based on foundation type | Feet |
| Average annual loss (AAL) | Average annual loss for building and contents | ${AAL}_{B/V_{R}}$/${AAL}_{C/V_{R}}$ | The integral of the economic loss of building/contents as a function of the annual probability of exceedance across the full range of probabilities | Calculated | Proportion of $V_{R}$ (%) |
|  | Average annual loss for use | ${AAL}_{U,months}$ | The integral of the economic loss of use as a function of the annual probability of exceedance across the full range of probabilities | Calculated | Months |
|  | Average annual loss for building, contents, and use | ${AAL}_{B\$}{/AAL}_{C\$}{/AAL}_{U\$}$ | The integral of the economic loss of building/contents/use as a function of the annual probability of exceedance across the full range of probabilities in absolute currency | Calculated | U.S dollars |
|  | Total average annual loss | ${AAL}_{T}$ | Summation of average annual loss for building, contents, and use | Calculated | U.S dollars |
